# Impact of Visual Game-Like Features on Cognitive Performance in a Virtual Reality Working Memory Task: Within-Subjects Experiment

**DOI:** 10.2196/35295

**Published:** 2022-04-28

**Authors:** Eric Redlinger, Bernhard Glas, Yang Rong

**Affiliations:** 1 Tokyo Institute of Technology Institute of Innovative Research / Koike & Yoshimura Lab Tokyo Japan; 2 Technical University of Munich Munich Germany; 3 Tokyo Institute of Technology Tokyo Japan

**Keywords:** HMD, working memory, gamification, cognitive training, serious game, game, cognitive activity, user performance, visual memory, cognitive, user performance, mobile phone

## Abstract

**Background:**

Although the pursuit of improved cognitive function through working memory training has been the subject of decades of research, the recent growth in commercial adaptations of classic working memory tasks in the form of gamified apps warrants additional scrutiny. In particular, the emergence of virtual reality as a platform for cognitive training presents opportunities for the use of novel visual features.

**Objective:**

This study aimed to add to the body of knowledge regarding the use of game-like visual design elements by specifically examining the application of two particular visual features common to virtual reality environments: immersive, colorful backgrounds and the use of 3D depth. In addition, electroencephalography (EEG) data were collected to identify potential neural correlates of any observed changes in performance.

**Methods:**

A simple visual working memory task was presented to participants in several game-like adaptations, including the use of colorful, immersive backgrounds and 3D depth. The impact of each adaptation was separately assessed using both EEG and performance assessment outcomes and compared with an unmodified version of the task.

**Results:**

Results suggest that although accuracy and reaction time may be slightly affected by the introduction of such game elements, the effects were small and not statistically significant. Changes in EEG power, particularly in the beta and theta rhythms, were significant but failed to correlate with any corresponding changes in performance. Therefore, they may only reflect cognitive changes at the perceptual level.

**Conclusions:**

Overall, the data suggest that the addition of these specific visual features to simple cognitive tasks does not appear to significantly affect performance or task-dependent cognitive load.

## Introduction

### The Emergence of a Cognitive Training Industry

The recent widespread availability of game-like cognitive training products in the form of *apps* on smartphones and tablets, along with a growing public awareness of cognitive training in general, have all contributed to the creation of a multibillion-dollar industry [1]. However, long before the first commercial *brain training* apps appeared on smartphone app stores, a series of widely publicized studies helped set the stage for broader public acceptance of cognitive training. In one such study from 2003, Bavelier and Green [2] documented an increased attentional capacity for players of action video games. Although such differences are easily dismissed as a result of innate abilities or self-selection (eg, individuals with these capacities tend to gravitate toward gaming), the authors notably demonstrated that similar capacities could also be acquired by previously *nongamer* participants through a simple training regimen derived from the same games [2]. Another early, influential training study that received mainstream exposure was that of Jaeggi et al [3]: “Improving fluid intelligence with training on working memory.” The authors documented significantly increased *fluid intelligence* (the ability to reason and solve new problems independently of previously acquired knowledge) after cognitive training using a working memory task [3]. The study subsequently received widespread media coverage in outlets such as Wired magazine. Finally, an ambitious, multisite, longitudinal study made additional news headlines in 2017 when it concluded that a kind of adaptive, speed-of-processing task known as *Useful Field of View (UFoV) training* resulted in a significantly decreased risk of dementia up to 10 years after the training intervention [4]. These studies, among others, were instrumental in increasing public awareness of the possibility that explicit training might yield cognitive dividends and contributed to the recent industry boom.

### Ongoing Controversy

However, outside the world of public opinion, the overall efficacy of cognitive training remains controversial. Proponents have demonstrated benefits ranging from better scores on standard cognitive assessment tests [5,6] and improved performance in driving aptitude tests [7] to general gains in memory, attention, and visual-spatial ability [8,9]. Nevertheless, recent studies that report little or no benefit from cognitive training, including screen-based training, also exist in substantial numbers [10-13]. The discrepancies in study results are variously attributed to a lack of agreement on experimental methodology, outcome assessment, and the design and implementation of the cognitive training tasks themselves [14,15]. Even simple deviations from the convention can potentially have a major impact on the results. For example, a recent study by Linares et al [16] found no evidence of a *near-transfer* effect (ie, performance improvements in related tasks following training), even between very similar working memory tasks. However, an inspection of their protocol revealed that the training task used in the study was nonadaptive (ie, task difficulty was not adjusted to match participants’ natural abilities or prior training gains). This detail may have negatively affected the study’s findings, as recent studies argue that adaptive training may be an *essential* component of the success of cognitive training [15,17]. In addition, environmental factors may have contributed to the lack of an observed effect in the Linares et al [16] study as the training sessions were unsupervised, but the assessment sessions were conducted by study staff, which is a source of social stress and a potentially confounding variable [18,19].

### Clinical Studies Versus Commercial Cognitive Training

Makers of commercial cognitive training apps regularly advertise their products as *proven effective* and *based on real science* [20]. Some have even licensed or repurposed the very tasks that were used in well-regarded clinical studies [21]. However, just as simple deviations from task design convention may risk negating training effects in clinical studies, the repackaging of promising cognitive training tasks for use in commercial applications must be carefully considered to minimize any risk of introducing new cognitive demands.

**Figure 1:**
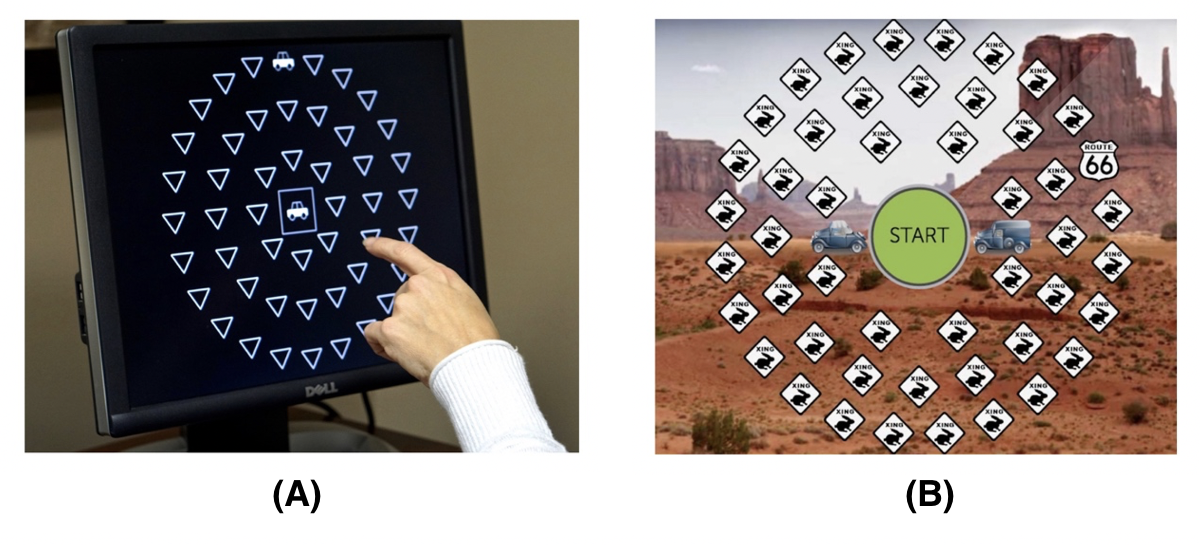
(A) Useful Field of View (UFoV) assessment test compared with (B) commercial cognitive training task dual decision (Posit Science Corporation) designed to train UFoV capacity. The task depicted on the right uses a similar, circular task design but includes colorful icons, scoreboards, and a game-like setting.

For example, one primary assessment outcome used in the 2017 study cited previously was the UFoV test. This test, which contains several subtests to assess short-term recall and spatial memory, requires participants to identify a previously displayed stimulus from among various similarly shaped distractors. Depending on the subtest, the stimuli may appear in either the central visual area, the peripheral area, or both. Although the original task, first developed in 1986, was designed as a clinical assessment tool for use with a standard monochrome computer monitor, commercial adaptations of the UFoV task generally add a number of additional visual and narrative elements to make the task more appealing to customers. These elements include the use of cartoon-like icons; colorful, task-irrelevant background imagery; thematic storylines; scoreboards; and others (Figure 1). Although the use of these elements has become commonplace in the commercial cognitive training industry, consensus on whether such modifications significantly affect the effectiveness of their core tasks is still elusive.

### A Closer Look at Gamified Tasks

Gamification is generally defined as the process of adding game elements to nonentertainment settings to increase motivation and engagement [22]. Game-like elements may broadly be considered to include visual elements such as colorful icons or patterns, evocative imagery, and playful animations, along with elements designed primarily to stimulate motivation, such as scoreboards and real-time performance feedback. When coupled with cognitive tasks specifically designed to maintain or improve one’s cognitive abilities, this result may be referred to as *gamified cognitive training*. The embrace of gamification as a method of increasing user engagement and enjoyment of otherwise dull, repetitive tasks is indeed supported by a significant number of studies [22-25]. However, the full picture of the potential impact of gamification on cognitive *performance* is less conclusive. Two recent comprehensive reviews [22,26] that examined the use of gamification strategies in brain training and general cognitive assessment studies overwhelmingly found that although gamified training appears to boost participant motivation, study heterogeneity impeded the drawing of clear conclusions with respect to performance or ecological validity (ie, the degree to which experimental results are generalizable to real-life situations). For example, the authors of the first study [26] identified no fewer than 28 game-like elements used in the 33 studies surveyed. These included positive and negative task feedback, time pressure, storylines or narrative elements, performance status displays, and many others. The second survey [22], from 2020, found that of the 49 papers examined, no study reported on the effect of a single element alone and that the game elements were investigated only in combination, making it impossible to establish whether individual elements had measurable effects.

For example, the 2017 study by Mohammed et al [24] compared two adaptations of an n-back task: a stripped-down task and one that contained a visually rich display combined with multiple audio soundtracks. Although the authors found increased task enjoyment for the game condition, there were no significant differences in the long-term outcomes between the gamified and nongamified tasks. However, given the complex set of features included in the gamified version, they acknowledged that more granularity was perhaps needed to fully understand which features might prove to be more successful than others [24].

Another study with a sizable participant pool (n=107) found negative correlations between certain game elements and task performance [27]. The authors speculated that unneeded stress and new cognitive demands might have been induced by distracting game elements such as persistent score displays, leading to reduced performance. However, rather than individual game elements added to a bare-bones task, the study design removed specific game elements from a larger group of game features. This approach seems to leave the possibility open for the remaining elements to compensate for the removal of a single element, making it difficult to know for sure which element or elements might have specifically accounted for the *new* cognitive demands [27].

In summary, as gamification encompasses a great number of individual elements, a lack of precision and homogeneity between studies has hampered the ability to draw consensus conclusions regarding which game elements, if any, may affect task performance. In addition, although motivational features such as scoreboards and real-time performance feedback have been widely studied [25,27-31], the specific impact of certain purely visual features, such as 3D depth and colorful, immersive backgrounds, is less well-documented, despite being increasingly encountered in consumer products such as game systems and dedicated virtual reality (VR) headsets.

Therefore, this study aimed to add to the body of knowledge regarding the use of game-like visual design elements by specifically examining the application of two particular visual features: immersive, colorful backgrounds and the use of 3D depth. These features were specifically chosen because of their underrepresentation in previous studies and their increased use in VR and augmented reality technology, a rapidly growing consumer market segment that also contains cognitive training products. We hypothesized that task performance may be adversely affected by additional visual processing demands but that the motivational effects documented by previous researchers may, in turn, compensate or reverse these effects. Finally, using electroencephalography (EEG) as an additional quantitative outcome, we hoped to gain insight into the possible neural correlates for any observed performance impact.

## Methods

### Study Design and Sample Size Considerations

Two primary outcomes were used to examine the impact of visual gamified design elements on cognitive task performance. Cognitive activity will be broadly measured along the midline using EEG (see the *EEG Data* section for details regarding EEG). Raw task performance was assessed by analyzing task accuracy and participant response time. The experimental task was a simple visual working memory task that required the participant to pick out the previously displayed stimulus from several distractors. To better control the testing environment, the task was coded for display in a head-mounted display (HMD) environment rather than a traditional monitor screen (see the *Experimental Task* section).

The use of an HMD serves two purposes: (1) to precisely control the display brightness and task visual angle (VA) across participants and experimental conditions and (2) to minimize potentially distracting external stimuli. For these and other reasons, several recent papers have recommended the use of HMDs, describing them as among the “most fitting platforms for applying nonpharmacological computerized neurocognitive assessments” [14] and a “frontier for neurorehabilitation” [32].

The current experimental task was previously used in a related study exploring changes in the size and position of visual stimuli and showed a robust effect size (>0.5) between conditions [33]. For this study, we undertook several additional modifications to further boost statistical power. First, to reduce between-subject variability, an adaptive task design was used in which task difficulty was automatically modulated to ensure maximum participant engagement. The precise method is described in more detail in the *Adaptive Task* section.

Second, an intrasubject protocol design exposed each participant to all experimental conditions. This enabled the use of repeated-measures ANOVA and Wilcoxon signed-rank sum tests, which are known to be particularly robust in establishing significance in small-n situations [34,35]. With this study design, we used the G-Power algorithm [36] to determine that a sample size of n=20 should be sufficient to enable us to achieve adequate statistical power at the 5% confidence level.

### Test Environment

A standalone HMD (HTC Vive Focus, HTC Corp) in its default configuration was chosen for the test environment. The cognitive training task was created in *Unity 3D*, a programming environment commonly used for creating 3D visual content for VR headsets (Unity 3D; Unity Software Inc).

HMD systems typically rely on handheld pointers for user input. However, such input devices are not appropriate for EEG studies, as they could introduce muscle-related artifacts. To address this, a touch screen smartphone was programmed to wirelessly send network commands to the HMD. A soft foam overlay with holes corresponding to the locations of the on-screen virtual buttons was added to the screen. With this combination, the participants could identify the smartphone controls in a tactile manner using only their hands without any need to view the screen. This is crucial as the participant cannot see the smartphone screen while wearing the headset.

**Figure 2:**
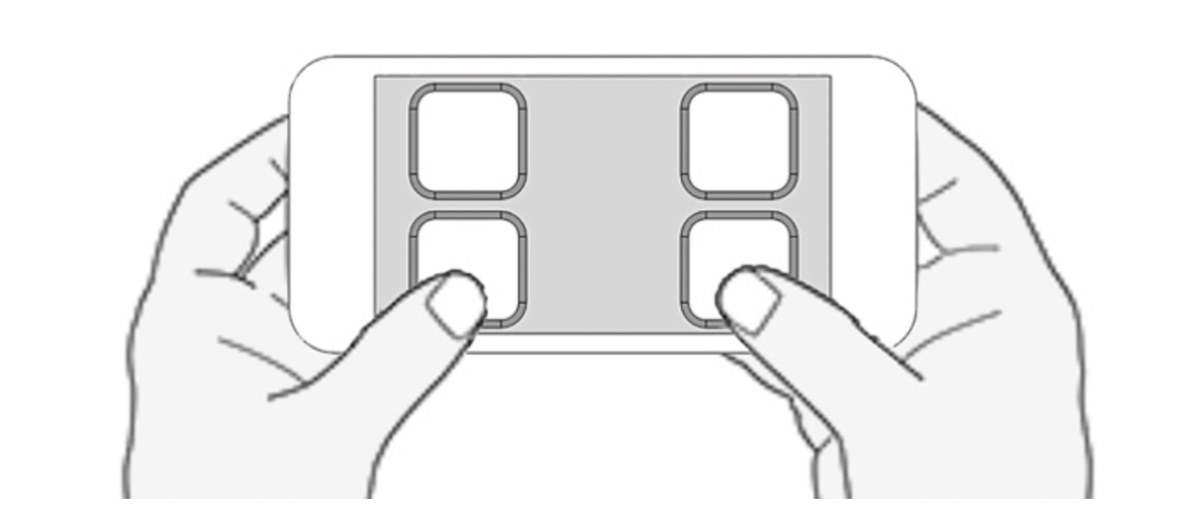
Smartphone interface with a foam overlay.

During the experiment, participants were seated and instructed to hold the smartphone controller in their laps, cradled by both hands (Figure 2). The experimental task was performed by tapping the virtual buttons on the screen with both thumbs while minimizing other body movements.

### Experimental Task

**Figure 3:**
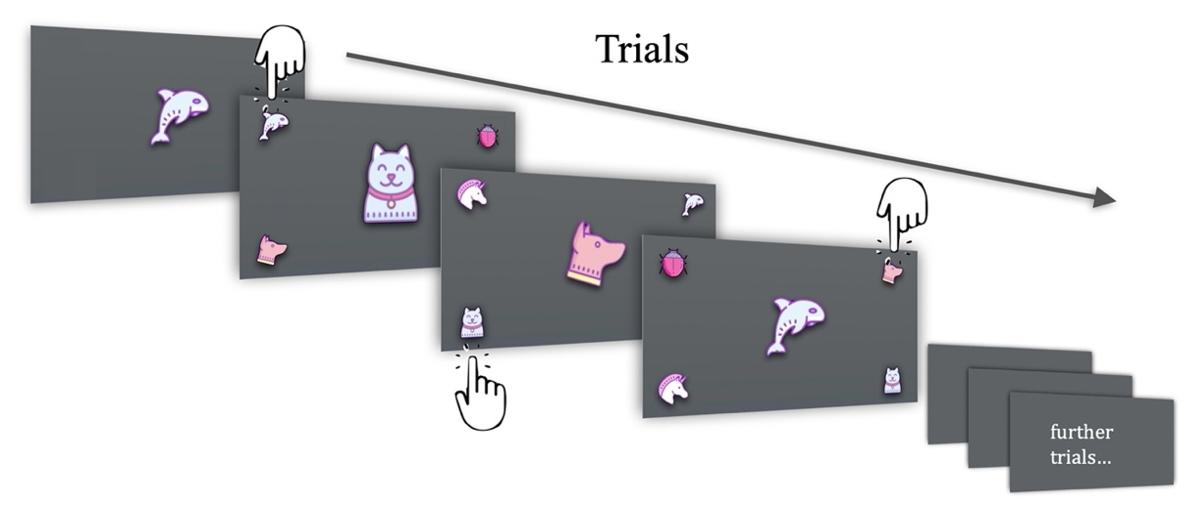
Sample trials showing the center stimulus and peripheral answer choices: the current answer choices (small images) and the next stimulus (large image) are displayed simultaneously. Preceding the first trial, only the initial stimulus is displayed. Participants select correct answers in the subsequent trials, as demonstrated with cartoon hands. In each case, the correct answer corresponds to the center stimulus from the previous slide.

To emulate a typical commercial cognitive training task, we designed our core task to incorporate a number of cognitive processes drawn from both gaming research [2,37] and the cognitive training literature [3,4,38]. These included visual memory recognition, divided attention, perceived time pressure, and distractor avoidance. The experimental task required participants to focus on a sequence of stimuli located in the center of the HMD screen. At the start of each new trial, the previously displayed center stimulus was moved to 1 of the 4 corners of the display, and a new stimulus took its place in the center. A total of 3 randomly chosen images were placed in the remaining 3 corners so that the screen always contained 1 center image and 4 images in the outer corners. To proceed to the next trial, the participant was asked to identify the stimulus that was *previously* in the center of the display. Participants performed this task by tapping the virtual button on the smartphone screen corresponding to the location of the object they wished to select. Once a choice was made by the participant, the answer choices disappeared, and the stimulus currently at the center of the display was reassigned to 1 of the 4 corners. A new stimulus then took its place in the center (Figure 3). A trial was also considered ended if the allotted time elapsed before a selection was made. Please see the *Adaptive Task* and *Experimental Protocol* sections for specific details related to trial times and durations.

In each trial, the center stimulus and incorrect answer choices were selected at random by the software such that no duplicate images appeared together. The trials lasted approximately 1.2 seconds (SD 116 milliseconds) on average and were designed to elicit continuous cognitive load as both the current answer choices and the following stimulus were displayed simultaneously. This was to minimize the usual *peaks and valleys* in the cognitive activity that often accompany tasks that alternate between stimulus presentation and participant response.

The goal of choosing this experimental task was to create a minimally complex task that could nevertheless reliably elicit sufficient cognitive load with little prior task training. Although the basic mechanism is inspired by the classic n-back task, we restricted our task to 1-back to minimize individual differences in performance ability commonly associated with higher degrees of *n* [39].

The figures themselves are from a set of 20 cartoon animal images, all drawn in a similar style but differing in shape and color. The image collection was licensed for noncommercial use from a popular internet vendor. It was chosen for its design similarity to prevailing commercial cognitive product designs, which frequently use a similar cartoon design aesthetic.

### Adaptive Task

**Figure 4:**
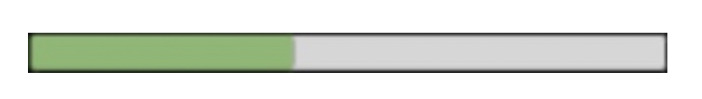
Adaptive task countdown timer.

An adaptive model was chosen for the experimental task to ensure similar engagement levels for all the participants. As the experiment progressed, the task difficulty increased incrementally until the participant failed to respond within the allotted time window or made ≥2 sequential mistakes. The task difficulty level was reflected in the amount of time available for the participant to choose an answer. As the difficulty level rose, this amount of time decreased in 50-millisecond intervals. Conversely, if the difficulty level decreased, more time (50 milliseconds) was made available to complete each trial. The prevailing task difficulty level affected the experiment in the following two ways:

1. A visible countdown timer just below the task area displayed the amount of time allocated to make a selection. As the trial time progressed, the bar’s contents filled incrementally from left to right, reminding the participant to answer as quickly as possible. The bar was purposefully designed to be as unobtrusive as possible so as not to distract from the primary task (Figure 4).
2. Failure to make a selection within the allotted time resulted in the trial being marked incorrect, and the next stimulus was presented. Making any selection (correct or incorrect) resulted in the timer pausing briefly (200 milliseconds) before being reset for the next trial.

At the end of each trial, the response (or failure to respond), the reaction speed, and accuracy were recorded. Only trials in which the participant actively made a selection were included in the reaction time assessment.

### Experimental Protocol

A total of 20 participants, aged 21 to 48 (mean 28.6, SD 7.7) years, were recruited from among students and staff at the Tokyo Institute of Technology and agreed to participate in the experiment after signing an informed consent form. The 20 participants included 6 (30%) women and 14 (70%) men, all right-handed, with no history of color vision disorders. In addition, all participants reported having had a previous experience using an HMD.

The protocol was executed in the following order: task training, EEG baseline activity measurement, and experimental conditions. The EEG baseline measurement phase (60 seconds) involved viewing a black background with open eyes to record nominal cognitive activity with no visual stimuli.

**Figure 5:**
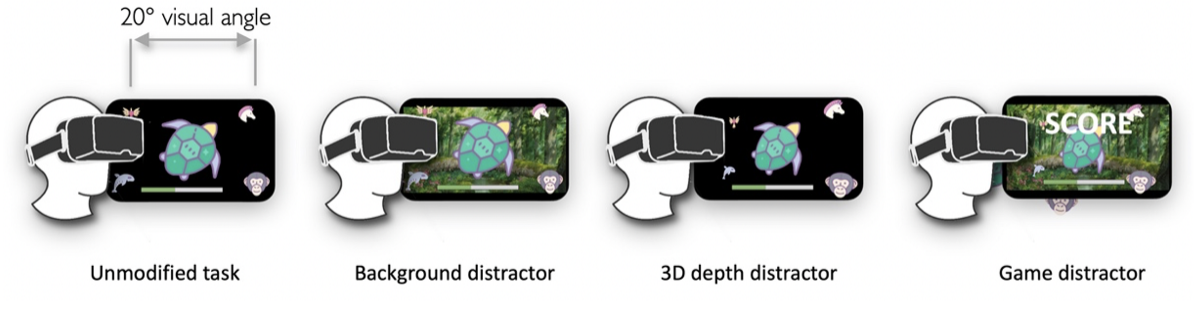
The four experimental conditions: the unmodified task on a black background; the task performed atop an irrelevant, colorful background; the task performed in 3D space; and the task with both background and depth distractors plus an interactive scoreboard and user feedback. Horizontal dimensions of core task limited to a 20º visual angle.

The experimental conditions comprised 4 distinct visual representations of the same core task: unmodified (the stimuli were simply placed on a flat plane against a black background), background distractor (stimuli + irrelevant background image), 3D depth distractor (stimuli presented at different virtual distances from the participant), and game distractor (dynamic motivational features in addition to the 2 previous distractors; Figure 5).

The image used in the *background distractor* condition was a cartoon forest scene obtained from the same provider as the stimulus images. The colors, detail level, and visual style were similar to those of the stimuli; however, there was no other obvious contextual connection. The game condition’s dynamic features comprised a scoreboard and real-time performance feedback. The performance feedback was implemented as follows: an incorrect user response caused the selected answer choice to briefly shake back and forth to indicate *no*, whereas a correct choice caused the item to gently pulse outward toward the user. These animations lasted exactly 200 milliseconds. In addition, a scoreboard at the top of the display indicated the current accuracy rate and total score for the current trial set.

All experimental conditions were repeated twice in a randomized order for a total of 8 sets per participant. Each set contained 50 trials and lasted approximately 60 seconds. A 30-second break (black screen; no visual stimulus) was imposed between the training and baseline phases. This was done to prevent contamination of the baseline EEG data by lingering arousal from training. Between each set of trials, there were additional 10-second rest breaks.

The task VA for all conditions was set at 20º, corresponding to the outer edges of the answer choices, measured horizontally. The VA was calculated using the following standard formula:

VA = (S × 57.29) / D **(1)**

Here, *S* is the size of the object, and *D* is the distance from the observer.

This VA was shown in a previous experiment to be optimal for maximizing the task training performance [33]. With the exception of the *3D depth distractor* and *game distractor* conditions, all visual task elements were precisely placed at a virtual distance of 2 m from the user, as viewed within the HMD. In the conditions that made use of 3D depth, the answer choices (and colorful background) remained at the same virtual distance of 2 m; however, the primary central stimulus moved forward to appear at a distance of 1 m from the user. In the Unity 3D programming environment, 1 unit of space is equivalent to 1 perceived meter of distance. To set the VA for each experimental condition, we specified the desired VA and solved the abovementioned equation for *S*. The value of *S* was applied to the visual task automatically by the software for each new experimental condition before the presentation of the first task trial.

Body movements, particularly eye movements, have a high possibility of introducing movement artifacts into the EEG data. Therefore, participants were instructed to blink and adjust their posture as needed during rest breaks but to refrain from doing so during the trial sets themselves.

**Figure 6:**
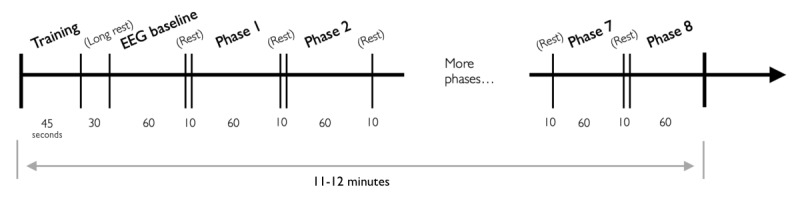
Protocol flow: following training and electroencephalograph (EEG) baseline recording, 8 experimental phases, each containing 50 trials, were conducted. A 10-second rest separated each experimental phase. The content of the experimental phases was randomly selected from the 4 condition types (unmodified task, background distractor, 3D depth, and game distractor) and balanced so that each participant experienced each condition twice. Unless otherwise noted, all times are in seconds; completion times for training and experimental phases are approximate. Total time to complete the protocol varied from 11 to 12 minutes per participant.

Visual text messages on display announced the beginning and end of these break periods. The latter message flashed off 2 seconds before the start of the following set. The total time required to complete each set of trials varied according to participant ability (as dictated by the rules of the adaptive task) but lasted approximately 60 (SD 7.49) seconds on average. This resulted in an overall experimental protocol duration of 11 to 12 minutes (Figure 6).

### Training

Before the start of the protocol, the task rules were explained, and each participant was granted time to practice the task until they were able to achieve a 75% average accuracy rate for at least 10 trials. Some participants mastered the task more quickly than others, such that the training period lasted between 30 and 90 seconds, with an average of 44 (SD 17) seconds. As the adaptive mechanism was also engaged during the training period, the training process also served to establish the starting difficulty for the participant for the following experimental trial sets.

### EEG Data

EEG signals (microvolts) were acquired from the frontal, central, occipital, and parietal regions using a wireless 8-channel EEG amplifier (OpenBCI 32-bit Board Kit, OpenBCI, Inc) with a sampling rate of 250 Hz. The electrode locations were Fz, Cz, Oz, and Pz, placed according to the international 10 to 20 system, and were specifically selected to capture a broad range of activity along the midline. In particular, we were interested in electrode positions Fz and Cz because of their frequently cited relationship with concentration and cognitive load, whereas Oz and Pz were chosen because of their proximity to the visual cortex and prior association with both attention and complex visual decoding [40-43]. Gold cup electrodes were attached to the scalp and ear lobes using an electroconductive gel, and an initial impedance of <5 kΩ across all electrode positions was ensured. Additional electrodes were affixed above and below the participants’ eyes to record electrooculogram signals caused by blinking or other facial movements for later use in noise reduction and signal optimization [44].

EEG data were recorded throughout the experiment, although only the final 30 seconds of activity were analyzed for each phase. This was to ensure that the task adaptation algorithm had been given sufficient time to adjust the difficulty levels for each participant before reaching the analysis time window. Time markers for determining the analysis epochs were embedded in the EEG data stream directly using real-time network packets generated by the experimental task. Through the use of this mechanism, we hoped to precisely measure similar levels of cognitive engagement for each participant.

### Task Performance

Overall reaction time and task accuracy were calculated for each phase and averaged across all trials for a given experimental condition.

### Analysis Method

The software used for EEG data preprocessing and analysis was MATLAB R2019b (MathWorks, Inc). The raw EEG data were notch filtered (50 Hz) and high-pass filtered at 4 Hz using built-in Butterworth and bandpass filters in MATLAB. As noted earlier, the electrooculogram data were recorded in tandem with the EEG for each participant. This enabled us to create customized artifact recognition routines that were individually applied during the data preprocessing phase for each participant. Additional muscle artifacts identified from a visual inspection of the EEG data plots were also removed in full from the time series before analysis.

Fast Fourier transforms were calculated for the following spectral ranges: theta (4-8 Hz), alpha (8-13 Hz), low beta (13-20 Hz), and high beta (20-28 Hz), with 30-second windows for each phase of the experiment. The total sum of the power values from each range was divided by the total number of EEG data samples. The resulting score was normalized by subtracting the overall population mean (combined EEG data of all participants divided by the number of participants) and dividing by the SD to obtain the power index. Fast Fourier transforms and statistical analyses were performed using built-in MATLAB functions.

Shapiro-Wilk tests showed that we could not necessarily operate under an assumption of normally distributed data. Therefore, statistical significance was determined with a repeated-measures ANOVA, followed by a nonparametric Wilcoxon signed-rank test to determine the significance of any changes in power between the experimental phases. The Wilcoxon test was chosen because of the large individual differences in performance observed among participants, nonnormally distributed data, and the within-subjects nature of the study.

Task performance data were averaged to obtain an overall accuracy and reaction time value for each participant per task condition. Individual results were averaged, and similar Wilcoxon signed-rank tests were conducted.

When looking at the preliminary data, it became quickly apparent that the performance levels varied significantly from participant to participant. Some individuals were able to complete the task quickly and accurately, whereas others struggled to respond and made frequent mistakes. This contributed to a large SD in the overall results, which could potentially complicate the drawing of meaningful conclusions. To address this, participants were additionally subclassified into *high-* and *low*-performance groups for further analysis. The selection criteria were based on the average overall task difficulty level achieved by each participant.

### Ethics Approval

The experimental protocol was approved by the ethics board of the Tokyo Institute of Technology (2019059).

## Results

### EEG Data

The presence of gamified visual features led to observable changes in the spectral power at all EEG locations. In particular, the occipital and parietal areas showed noticeable increases in beta EEG power for the 3D depth distractor condition and in the theta rhythm during the background distractor condition. Overall, 1-way repeated-measures ANOVA showed significant differences in the high-beta range for all electrodes tested (Fz: *F*_3,76_=3.75, *P*=.02; Cz: *F*_3,76_=4.09, *P*=.01; Pz: *F*_3,76_=2.82, *P*=.046; Oz: *F*_3,76_=2.97, *P*=.04). Post hoc Wilcoxon signed-rank tests revealed that with the exception of the *game* condition at Fz, all individual increases in the high-beta rhythm between the unmodified and experimental conditions were significant at the 5% confidence level. However, the differences between individual experimental conditions were not significant.

**Figure 7:**
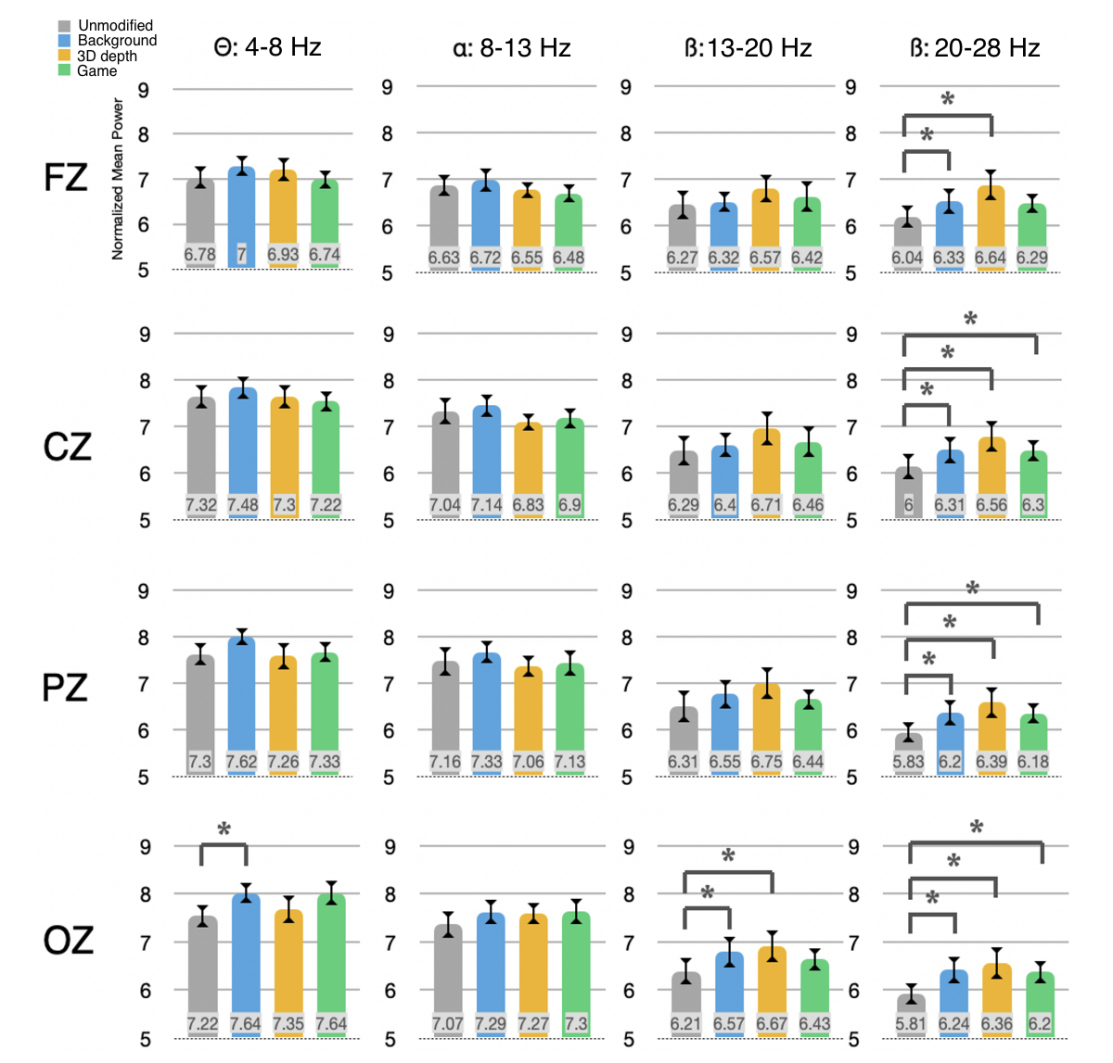
Spectral power by condition and frequency at electroencephalograph locations Fz, Cz, Pz, and Oz; n=20, SE; statistical significance calculated with Wilcoxon signed-rank test (*P<.05).

In contrast, for the theta range, only the results at Oz displayed significant variation (*F*_3,76_=3.20; *P*=.03), and only one individual experimental condition, the background distractor, proved to be significant (n=20; *Z*=−2.81; *P*=.00495) in the post hoc analysis. Changes in the alpha rhythm did not prove to be significant at any electrode position (Figure 7).

It is noteworthy that the game condition, which also included the 3D depth distractor, did not reach the same levels of cognitive activity as the depth-only condition for the beta range. This may indicate that the presence of additional distractions in the game condition inhibits the overall impact of the 3D depth effect. However, in the theta range, the presence of background distraction in both the background and game conditions led to similar cognitive responses.

### Performance Data

**Figure 8:**
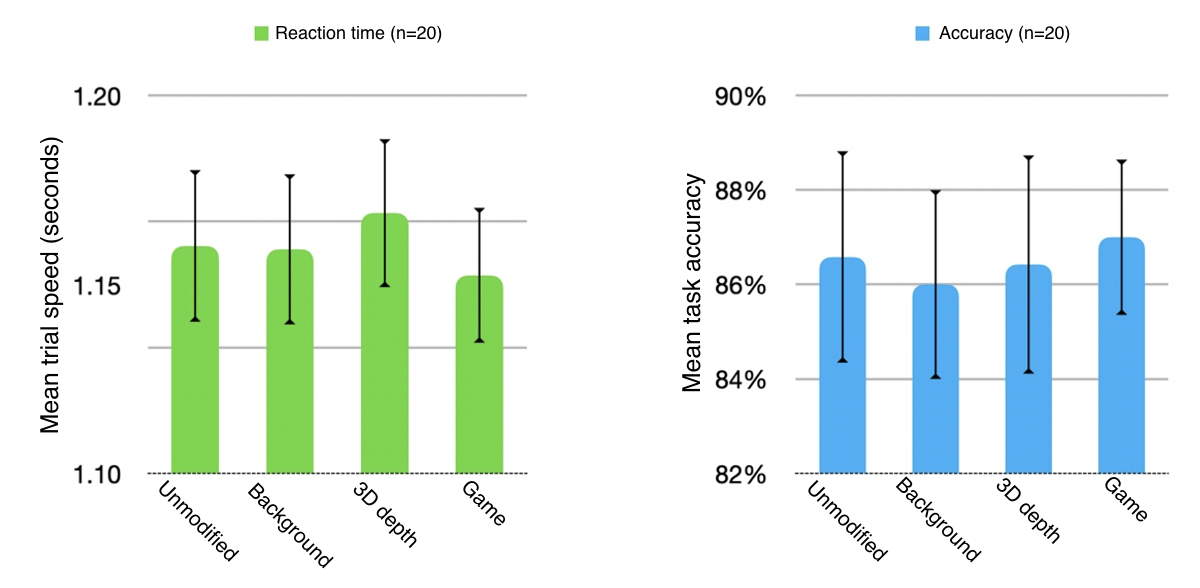
Mean task performance (reaction time and accuracy) by condition and group; SE; statistical significance calculated with Wilcoxon signed-rank test (*P*<.05).

The 1-way repeated-measures ANOVA comparing the 4 conditions showed no significance for either task speed (*F*_3,72_=1.21; *P*=.31) or accuracy (*F*_3,72_=0.143; *P*=.93). In general, the presence of colorful, task-irrelevant backgrounds led to slight reductions in accuracy but had little impact on performance speed. Conversely, the presence of 3D depth cues seems to have slightly affected reaction time but not accuracy (Figure 8).

As noted previously, we took the additional step of separating participants into high- and low-performance groups according to ability (average maximum task difficulty achieved during all trial sets) as a supplemental analysis. This was because of a large SE observed in the performance data, which we felt had the potential to mask underlying trends. Although the resulting subgroups were too small to deliver meaningful statistical power, the results revealed several nuances and presented a potentially interesting direction for a follow-up investigation.

For task accuracy, the additional visual distractions present in the multiple-distraction game condition appear to have had a cumulative negative impact on high performers. However, a seemingly opposite effect was observed in the low-performance group, which cumulatively achieved the highest accuracy in this condition.

**Figure 9:**
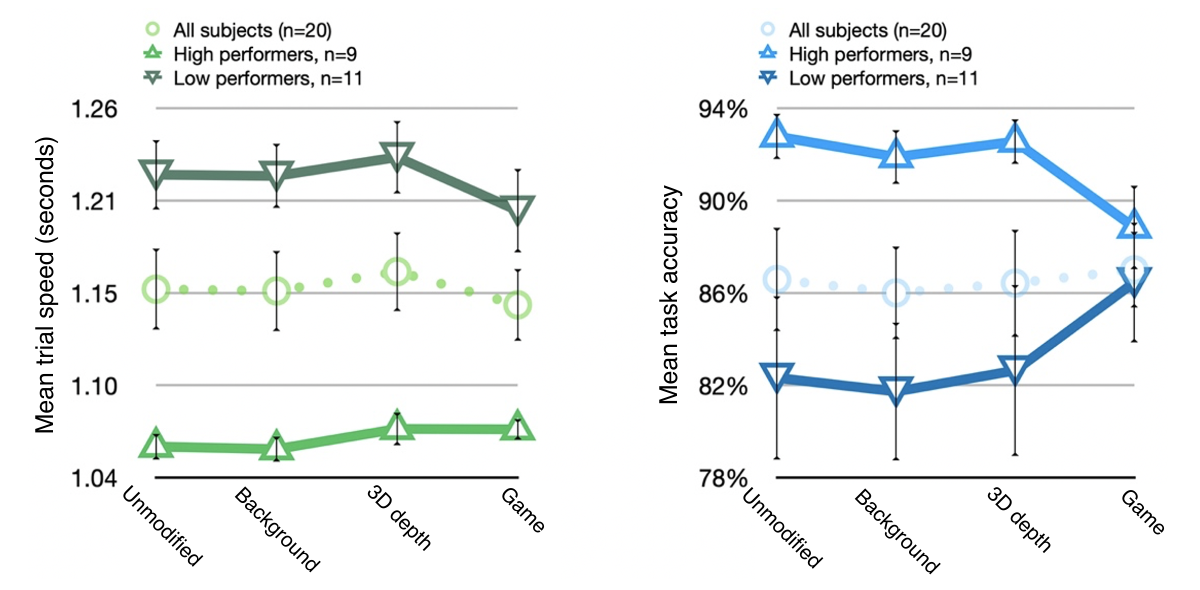
Mean task performance (reaction time and accuracy) by condition and high- and low-performance subgroup; SE.

In terms of task completion speed, our results did not show any significant differences between conditions, even when observing only the more internally homogenous high-performance subgroup (Figure 9).

### Performance and EEG Compared

Perhaps because of a lack of significant differences in performance between experimental conditions, regressing EEG spectral power onto performance results produced no meaningful correlations for either the overall group or either of the subgroups. Large individual differences in participant performance likely also contributed to the lack of significant results.

## Discussion

### Principal Findings

The objective of this study was to examine the impact of visual, game-like elements on task performance and cognitive activity in a visual working memory task. No significant differences in performance could be determined for both reaction time and task accuracy. Nevertheless, certain performance trends can be observed that seem to leave open the possibility that specific types of visual distractions may affect some aspects of cognitive performance while leaving others unaffected. For example, our data show that visually distracting backgrounds had no observable impact on reaction speed but had a slight impact on accuracy. Conversely, 3D depth decoding appears to have slightly affected the speed of processing but not the task accuracy.

Similarly, the EEG power analysis revealed no significant differences in the crucial frontal theta rhythm at Fz, which often serves as a proxy for participant concentration and task engagement [41,43]. In contrast, significant differences between conditions were observed in the beta band and theta band at the occipital electrode. Although these results return to insignificance if one corrects for multiple comparisons using Bonferroni or a similar method, the question nevertheless arises as to what might have caused these observed effects in the beta and theta rhythms, particularly given the lack of correlation with performance. For instance, the higher theta power observed at Oz was actually accompanied by slightly *reduced* accuracy in the background distractor condition. The proximity of Oz to the occipital area and the visual cortex suggests that rather than being directly linked with cognitive effort related to the task, perhaps the theta rhythm is simply more sensitive to certain underlying ocular processes required by the visually rich background used in this condition.

For example, although the current experimental task is designed to prohibit voluntary eye movements by requiring the participant to continually focus on a center stimulus, the presence and frequency of *involuntary* eye movements such as saccades were unfortunately not recorded as part of the current experimental design. Indeed, evidence suggests that saccades may be highly correlated with theta power during periods of memory encoding [45]. Other studies have similarly observed links between increased cognitive stress related to memory tasks and elevated saccadic frequency and duration [46,47]. Thus, the possibility that the background condition may have elicited a disproportionate amount of ocular activity and, along with it, increased theta power represents one hypothesis for the observed results.

At the same time, increased high-beta (20-28 Hz) spectral power in the 3D depth condition was accompanied by generally slower reaction times. Although previous research has implicated beta rhythm in a variety of assistive roles with regard to visual perception [48], studies that specifically examine 3D decoding are less conclusive. For example, although some researchers found that 3D environments elicited greater cognitive activity than their 2D counterparts, particularly in the beta range [49], Dan et al [50] found a *reduction* in EEG power during the 3D condition versus the 2D condition in their experiment involving a learning task [50]. However, the latter study involved complex *reality-like* visuals, focused on the Fz theta/Pz alpha ratio for EEG feature classification rather than a broad-spectrum analysis, and did not specifically target the beta range. Therefore, the possibility remains that, as with the theta band, underlying cognitive demands related to visual processes may have obscured task-related cognitive activity. As noted earlier, the cognitive task used incorporates several cognitive processes, including visual working memory and divided attention. This multimodality presents a further challenge when trying to determine the exact reason for unexpected EEG results, as it is difficult to ascertain the cognitive process responsible for the observed effects.

The supplementary analysis of performance by participant ability, although not statistically meaningful, nevertheless revealed an unexpected trend with regard to task accuracy. The performance results from the high group appeared to be cumulatively reduced by successive layers of distractions, with the game condition eliciting the lowest average accuracy levels. The poorer performers paradoxically appeared to perform best during this condition. However, it must be noted that the average degree of accuracy obtained in the *low* group was still well below that of the average overall performance from the *high* group.

We offer two hypotheses: throughout the experiment, the low-performance group may have experienced a form of performance anxiety that led to generally slower decision-making and lower overall accuracy. However, the presence of multiple additional visual elements in the game condition may have provided a certain degree of reassurance and encouragement, an effect of gamified design documented by previous researchers [14,25]. Similarly, the inclusion of a scoreboard and positive and negative response feedback after every trial in the game condition may have helped to refocus participant attention and encourage less experienced or more easily distracted participants to improve their performance.

Finally, it is worth noting the limitations of the current results. First, as the context of this study was potential users of commercial cognitive training products, we used broadly inclusive criteria for participant selection, which resulted in a wide range of ages and an uneven gender balance. This may have affected the results in unexpected ways. Second, although all experimental conditions differed significantly from the unmodified task in the high-beta range (except for the game condition at Fz), they did not differ significantly from each other. This lack of precision reinforces the possibility that any visual novelty, whether it is the presence of 3D depth or a colorful background, triggers an increased cognitive response in the high-beta range. Greater EEG channel density and separating the multimodal task into its component cognitive processes could potentially help isolate and differentiate the observed responses.

### Conclusions

In isolation, a small performance impact was incurred by the inclusion of a colorful, task-irrelevant background and the use of 3D depth elements. However, that impact was mitigated or reversed for some participants when combined with *motivating* features such as real-time feedback and scoreboards. Overall, the primary finding of this study is that performance in simple memory tasks of the kind that are frequently found in commercial cognitive training apps is not significantly affected by the use of visually distracting backgrounds or 3D depth or by common motivational game elements such as scoreboards and real-time performance feedback. Particularly in light of the user engagement and motivational advantages of gamification documented by previous researchers, the observed impacts may not be substantial enough to warrant specific design patterns or the redesigning of existing gamified cognitive tasks unless the specific goal is to maximize the speed and accuracy, in which case, the current findings may provide some useful guidance.

## Abbreviations

ACTIVE: Advanced Cognitive Training for Independent and Vital Elderly
EEG: electroencephalograph
HMD: head-mounted display
UFoV: Useful Field of View
VA: visual angle
VR: virtual reality
